# Factors influencing childbirth fear among Asian women: a scoping review

**DOI:** 10.3389/fpubh.2024.1448940

**Published:** 2025-01-14

**Authors:** Aida Kalok, Ixora Kamisan Atan, Shalisah Sharip, Nazarudin Safian, Shamsul Azhar Shah

**Affiliations:** ^1^Department of Public Health Medicine, Faculty of Medicine, Universiti Kebangsaan Malaysia, Kuala Lumpur, Malaysia; ^2^Department of Obstetrics & Gynaecology, Faculty of Medicine, Universiti Kebangsaan Malaysia, Kuala Lumpur, Malaysia; ^3^Department of Psychiatry, Faculty of Medicine, Universiti Kebangsaan Malaysia, Kuala Lumpur, Malaysia

**Keywords:** Asia, childbirth, fear, pregnancy, tokophobia

## Abstract

Fear of childbirth (FOC) or tokophobia adversely affects women during pregnancy, delivery, and postpartum. Childbirth fear may differ across regions and cultures. We aimed to identify factors influencing the fear of childbirth among the Asian population. A systematic literature search was performed using the PubMed, Scopus, and Web of Science databases in November 2023. Original articles in English with research conducted in Asian countries were included. The independent factors associated with childbirth fear, from the relevant studies were identified and discussed. Forty-six papers met the eligibility criteria but only 26 studies were discussed in this review. The significant factors were categorized into (1) demographics, (2) clinical, (3) healthcare service, (4) childbirth education & information, and (5) COVID-19 pandemic. The prevalence of childbirth fear among Asians ranged between 56.6 and 84.8%. Significant demographic factors included age, education, marital status, economic status, and area of residence. Greater levels of tokophobia were linked to nulliparity, unplanned pregnancy, infertility, miscarriage, and pregnancies at risk. Effective doctor-patient communication and more frequent antenatal visits were shown to alleviate maternal childbirth fear. There was consistent evidence of prenatal childbirth education’s benefit in reducing FOC. The usage of smartphone apps and prolonged exposure to electronic devices were linked to a higher degree of tokophobia. Nulliparas who received too much pregnancy-related information also reported increased childbirth fear. There was a positive correlation between maternal fear of COVID-19 infection and FOC. Keeping updated with COVID-19 information increased the maternal childbirth fear by two-fold. In conclusion, a stable economy and relationship contribute to lesser childbirth fear among Asian women. Poor maternal health and pregnancy complications were positive predictors of FOC. Health practitioners may reduce maternal childbirth through women’s education, clear communication as well as accurate information and guidance to expectant mothers. Further study is required into the content of childbirth fear among Asian women. These research findings hopefully will lead to the development of culturally adapted screening tools and interventions that reduce the burden of FOC among expectant mothers.

## 1 Introduction

Pregnancy and childbirth are among the most important events in a woman’s life. Fear of childbirth (FOC) or Tokophobia has been defined as “an unreasoning dread of childbirth,” and further classified into primary (affecting nullipara) and secondary (involving parous women who have experienced birth) (1, 2). A systematic review by O’Connell et al. in 2016 showed that the tokophobia prevalence ranged between 3.7 and 43% with a pooled prevalence of 14% (95%CI 12–16%) (3). Factors including anxious personality, traumatic delivery, previous miscarriages, low social support, and poor partner relationships have been associated with tokophobia (4, 5).

Childbirth fear has been associated with greater use of epidural, prolonged labor, and increased risks of labor dystocia as well as emergency cesarean section (6, 7). Previous studies demonstrated a link between tokophobia with negative birth experiences and post-traumatic stress disorder (PTSD) (4, 5). A negative childbirth experience can lead to dysfunctional maternal–infant bonding and reduced exclusive breastfeeding (8). Negative experiences also carry a greater risk of postpartum depression and negatively impact individual’s attitudes toward future birth, resulting in a maternal request for cesarean delivery (9, 10). Post-partum PTSD adversely affects not only maternal psychological well-being but also the child’s socioemotional and cognitive development (11). Women with low economic status are at greater risk of PTSD and those from developing countries are particularly vulnerable due to healthcare limitations and stigma surrounding mental health (12).

Fear of childbirth is multi-dimensional. Qualitative studies demonstrated that women report various childbirth concerns including maternal and child risk, loss of control, uncertainty, pain, and isolation (13). Understanding FOC in a local cultural context is paramount as women’s perception of childbirth is also strongly influenced by their culture (13).

There has been increasing interest in tokophobia in empirical research and clinical practice over the last 30 years (3). However, the lack of international consensus on the definition and gold standard measurement of tokophobia resulted in significant heterogeneity among the published studies (3). A review by Richens et al. found that a majority of research was conducted in Scandinavia and utilized a range of assessment tools to measure childbirth fear. The authors concluded that the inconsistent instruments used by the various studies were a reflection of the challenges in defining FOC (14).

Standardized tools to assess maternal childbirth fear may be restrictive due to a wide range of cultural backgrounds, alongside different perceptions and beliefs regarding birth (14). The Wijma Delivery Expectancy Questionnaire Part A (WDEQ-A) is the most commonly used diagnostic tool (15). WDEQ-A consists of a 33-item self-assessment questionnaire with a six-point Likert scale response per item, and a score ranging from 0 to 165. WDEQ-A score of ≥85 is indicative of severe fear of childbirth (16). It is a valid and reliable instrument in a variety of populations, while some concerns have been raised about the items’ cross-cultural applicability (17). Previous research demonstrated significant differences in the exploratory factor analyses of the WDEQ-A in European cohorts, suggesting that the content of FOC may differ between countries (18). Lukasse et al. demonstrated that FOC consisted of six factors, i.e., *Lack of self-efficacy, Loneliness, Negative appraisal, Lack of positive anticipation, Fear,* and *Concern for the child*. The prominence of each component varied across nations. For example, Icelandic women expressed the least lack of self-efficacy and recorded the lowest score for the fear component. Danish women had the least expectations of being lonely, while Belgian mothers scored the highest for the factor (18).

The majority of studies on tokophobia are conducted in developed countries from Europe, Scandinavia, and North America (19). As birth is a multifaceted experience, it is natural for childbirth fear to differ across regions or cultures (17). Recognizing the factors associated with tokophobia is essential in planning and providing an effective intervention to lessen its burden on expectant mothers. Our study aims to identify the factors which influence maternal childbirth fear among pregnant women in Asia. We hope that the findings will improve the understanding of FOC among Asian women and encourage more culturally adapted research on tokophobia.

## 2 Materials and methods

### 2.1 Literature search

This review was conducted according to the Preferred Reporting Items for Systematic Reviews and Meta-Analyses (PRISMA) guidelines for scoping review (20). We performed a literature search on electronic databases (PubMed, Scopus, and Web of Science) using the following search string; (childbirth fear OR “tokophobia” OR “prenatal fear”) AND (risk OR pred* or “risk factor”). The search frame was from the inception of the databases until September 2023.

Original articles in English and from Asian countries, with the following features, were included: (1) studies assessing childbirth fear as the main outcome; (2) childbirth fear assessment during the antepartum period or labor; (3) FOC was measured using a validated scale, and not as a subdomain of a larger assessment tool. We also included all cross-sectional and cohort studies alongside meta-analysis and systematic reviews to identify relevant studies. Articles with the following characteristics were excluded: (1) conference abstracts, letters or commentary, editorial and book chapters; (2) studies involving Asian immigrants in non-Asian countries or Asian-Americans; (3) studies conducted in the postpartum period; (4) qualitative or intervention study.

Endnote (version 20.6, Clarivate, United States) was utilized to organize the literature and detect duplicates. The article titles and abstracts were screened by two authors, A.K. and I.K.A. Subsequently, full texts were obtained for in-depth analysis following the inclusion and exclusion criteria. The third author (S.S) would be consulted to resolve any discrepancies in the study inclusion. The data extracted include authors (years), study design including assessment tools, subject characteristics (gestation, parity, pregnancy risk), and study findings (associated factors and correlations).

## 3 Results

**Figure 1:**
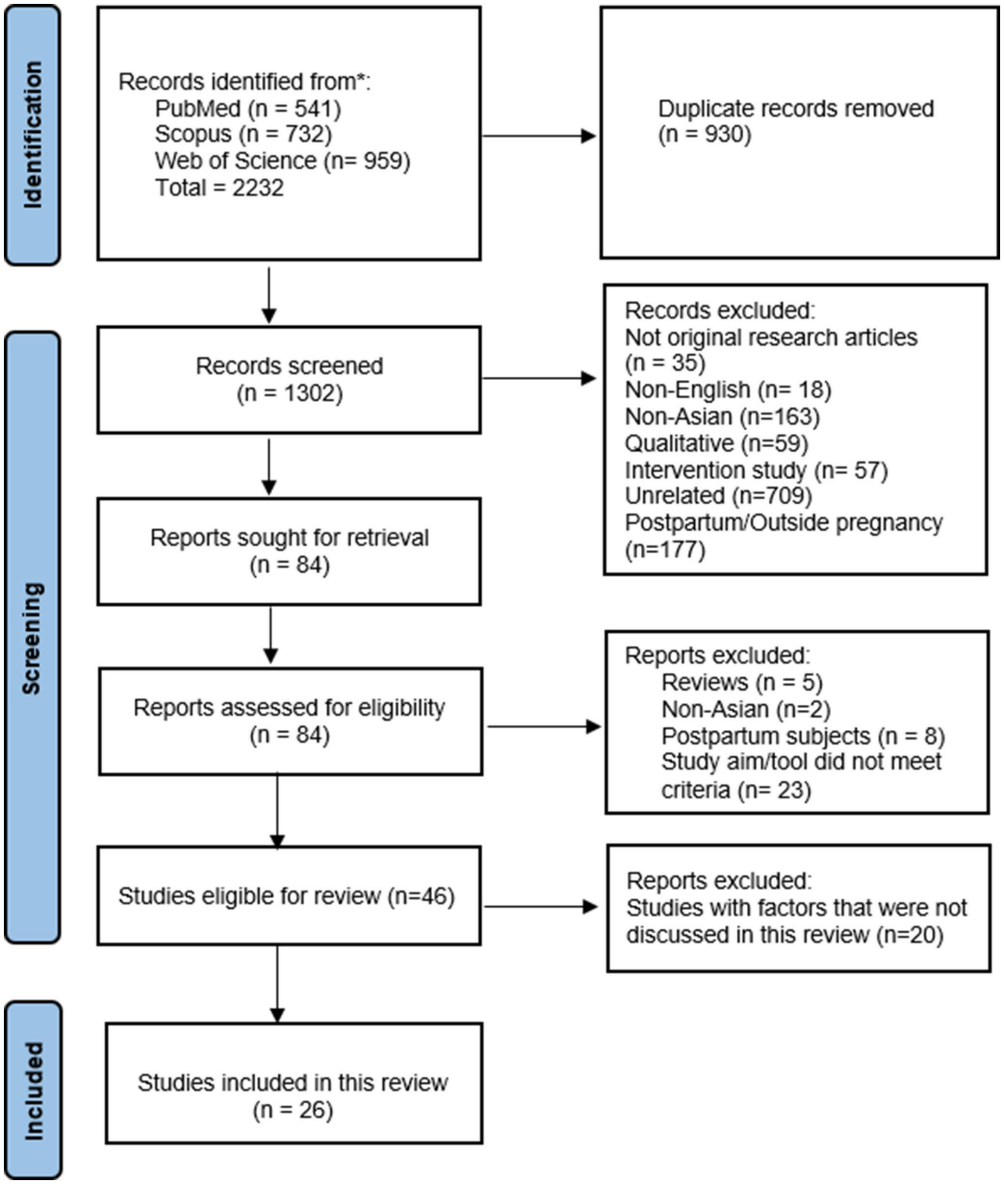
PRISMA flowchart demonstrating selection of articles.

The literature search using three electronic databases discovered 2,232 items (Pubmed = 541, Scopus = 732 and Web of Science = 959). After the removal of duplicates (930), we screened the title and abstract of 1,302 items. A total of 1,218 papers were excluded for various reasons resulting in 84 remaining items that were subjected to full-text screening. Finally, we identified 46 eligible papers for this review. Upon detailed assessment of the data, we found that the factors contributing to childbirth fear can be classified into several categories including (1) demographics, (2) clinical, (3) psycho-social, (4) healthcare service, (5) childbirth education & information, and (6) spiritualism. Due to the vast amount of data collected, we have decided to limit our discussion to the maternal demographic and clinical characteristics, health service provision, childbirth education, and COVID-19 pandemic; which involved 26 articles (Figure 1). The remaining findings related to the psycho-social and spiritual components were reported in a separate journal article.

Table 1 depicts the studies included in our review. Most of the research, which were published between 2014 and 2023, were conducted in Turkey. Our review included six studies from China and four from Iran. The remaining studies were from Japan, Vietnam, Thailand, Pakistan and Israel. A total number of 11,699 women were involved in all of the studies, which were of cross-sectional design. Majority of the studies used WDEQ-A or Childbirth Attitudes Questionnaire (CAQ) (21) as the assessment tool for childbirth fear. Four studies focused on nulliparous subjects, two on high-risk women and one study involved pregnant adolescents.

**Table 1:** Studies included in the review.

No	First author	Year	Country	Study aim: to investigate	Gestation in weeks; parity (special risk)	Total number (N)	FOC assessment tool	FOC Prevalence (level)
1	Aksoy (37)	2023	Turkey	The relationship between COVID-19 obsession & anxiety and FOC in high-risk pregnancies	≥20; all (high risk women)	326	FOBS	na
2	Anjum (49)	2022	Pakistan	The relationship among women’s fear of childbirth, well-being and partner support.	>35; nullip	100	WDEQ-A	na
3	Barat (27)	2023	Iran	To screen for FOC and associated factors	≥ 20; all	600	WDEQ-A	29.2% (severe)
4	Beiranvand (30)	2017	Iran	The prevalence of FOC and its associated factors in primigravid women	18–32; nullip	400	CAQ	80.8% (overall)
5	Buldum (57)	2021	Turkey	The relationship between social support and FOC in adolescent pregnancy.	≥28; all (adolescents)	100	WDEQ-A (Turkish)	na
6	Citak (17)	2021	Turkey	The psychosocial predictors of FOC in pregnant women.	≥28; all	624	WDEQ-A (Turkish)	20.8% (severe)
7	Eroglu (28)	2022	Turkey	The prevalence of FOC & associated factors including vaginismus in pregnant women with high/severe FOC	24–40; all	407	WDEQ-A (Turkish)	82.1% (overall) 13.8% (severe)
8	Gao (24)	2015	China	The FOC & its predictors among Chinese women	≥ 28; all	353	CAQ (Chinese)	na
9	Han (31)	2022	China	The associations between coping styles, intolerance of uncertainty, and FOC.	24–40; all	969	CAQ Chinese	67.8% (overall) 4.0% (severe)
10	Huang (25)	2021	China	The prevalence and predictors of FOC among Chinese Women	≥11; all	646	CAQ Chinese	na
11	Kaya (34)	2020	Turkey	The affecting factors and childbirth fears of Turkish pregnant women	≥28; all	78	WDEQ-A	20.5% (severe)
12	Kizilirmak (77)	2022	Turkey	The effect of childbirth-related Internet use by pregnant women on FOC	20–40; all	350	WDEQ-A	na
13	Matinnia (66)	2014	Iran	The content of maternal fear and the associated demographic factors	13–15; nullip	342	Fear Related to Pregnancy and Childbirth Questionnaire	48.2% (severe)
14	Moghaddam Hossieni (59)	2017	Iran	The prevalence of intimate partner violence (IPV) and its prediction of FOC.	≥14; all	174	Revised version of the Fear of Vaginal Delivery questionnaire (rFDQ)	61.5% (severe)
15	Nguyen (26)	2021	Vietnam	The FOC and willingness to pay for fear-prevention services in pregnant women	ns; all	900	FOBS	na
16	Ogurlu (55)	2023	Turkey	The effect of intimate partner violence on childbirth fear of pregnant women	≥28; all	335	WDEQ-A	84.8% (overall) 29.9% (severe)
17	Phunyammalee (42)	2019	Thailand	The prevalence of FOC in uncomplicated pregnant women & associated factors	28–36; nullip	305	WDEQ-A Thai	81.6% (overall) 0.7% (severe)
18	Qiu (29)	2019	China	The status of FOC and its associated factors among nulliparous women in China	ns; all	1,039	CAQ Chinese	na
19	Soysal (53)	2020	Turkey	The level of FOC childbirth in pregnant women and associated factors	32–36; all	444	WDEQ	75.2% (overall) 25.0% (severe)
20	Takegata (60)	2014	Japan	The relationship between FOC and Sense of Coherence (SOC)	37; all	226	WDEQ-A (Japanese)	na
21	Taubman (33)	2021	Israel	The effect of self-compassion, perceived social support and COVID-19 related childbirth anxiety on FOC	ns; all	403	PRAS	na
22	Tiryaki (38)	2022	Turkey	The fear of birth and COVID-19 in high-risk pregnant women	20–40; all (high risk women)	238	FOBS	na
23	Ulu (39)	2022	Turkey	The relationship between fear of childbirth, fear of Covid-19, and marital adjustment	ns; all	382	WDEQ-A	na
24	Yildrim (36)	2023	Turkey	The influences of anxiety and depression on FOC	≥ 28; all	501	WDEQ-A	72.7% (overall) 12.4% (severe) *
25	Zhang (36)	2023	China	The prevalence and risk factors of FOC among pregnant women in the third trimester of pregnancy	≥28; all	535	CAQ Chinese	56.6% (overall) 3.9% (severe)
26	Zhou (32)	2021	China	The prevalence and risk factors for fear of childbirth	14–41; all	922	CAQ	70.3% (overall) 6.0% severe
						11,699		

*WDEQ classification adjusted for consistency; na, not available; ns, not specified; nullip, nulliparous; multip, multiparous. CAQ, Childbirth Attitudes Questionnaire; FOBS, Fear of Birth Scale; PRAS, Pregnancy-Related Anxiety Scale; WDEQ-A, Wijma Delivery and Expectation Scale-A.

The overall prevalence of childbirth fear in our review ranged between 56.6 and 84.8%. This finding could be attributed to the different FOC assessment tools, with varying cut-off scores to define severity levels. Some authors quoted the overall FOC prevalence while some focused on severe tokophobia. Studies with WDEQ-A used a score of ≥38 as indicative of FOC and ≥ 85 as severe fear (22), while CAQ scoring to detect tokophobia and the severe level was ≥28 and ≥ 52, respectively (23). Research from Iran and Turkey in West Asia used four types of questionnaires (WDEQ-A, CAQ, Fear Related to Pregnancy and Childbirth Questionnaire, and the revised version of the Fear of Vaginal Delivery questionnaire). Data from East Asian countries like Thailand and China were based on WDEQ-A and CAQ.

We identified independent factors associated with fear of childbirth, based on multivariable logistic or regression analysis, as demonstrated in Table 2. The factors with a significance value of *p* < 0.001 are highlighted in bold. Factors that significantly correlated to FOC were also discussed in this review.

**Table 2:** Independent factors associated with childbirth fear.

Characteristics	Independent factors	Studies
Demographics		
Age	Maternal age (*β* = −0.14, *p* = 0.002)	Gao 2015 (24)
	Younger maternal age group (*β* = −0.175, *p* = 0.001)	Huang 2021 (25)
	**Maternal age** (*β* = −0.109, *p* < 0.001)	Citak 2021 (17)
	Age of partner (*β* = −0.10, *p* < 0.01)	Nguyen 2021 (26)
Education	Higher education: Diploma Education (AOR 1.93, *p* = 0.010); College education (AOR 3.27, *p* = 0.001)	Barat 2023 (27)
	Higher education levels (*β* = 2.06, *p* = 0.010)	Qiu 2019 (29)
	**Education level** (*β* = 0.085, *p* < 0.001)	Citak 2021 (17)
	Education (years) (AOR 1.12, *p* = 0.004)	Eroglu 2022 (28)
Economy	Husband’s employment (AOR 0.42, *p* = 0.007)	Barat 2023 (27)
	Lack of sufficient income (AOR 3.70, *p* = 0.020)	Beiranvand 2017 (30)
	Part time employment (*β* = 0.141, *p* = 0.004)	Han 2022 (31)
Marital status	**Marital status** (*β* = −0.058, *p* < 0.001)	Zhou 2021 (32)
Place of residence	Rural area (*β* = −0.088, *p* = 0.002)	Zhou 2021 (32)
Clinical		
Parity	**Multipara** (*β* = −0.135, *p* = <0.001)	Huang 2021 (25)
	Multipara (*β* = −0.11, *p* < 0.05)	Taubman 2021 (33)
	**Multipara** (*β* = −0.186, *p* < 0.001)	Han 2022 (31)
	Multipara (*β* = −1.64, *p* < 0.049)	Zhang 2023 (36)
Gestational age	Second trimester [vs third trimester] (*β* = −0.065, *p* = 0.009)	Huang 2021 (25)
	Gestational age (*β* = 0.80, *p* = 0.003)	Zhou 2021 (32)
Pregnancy planning	**Unplanned pregnancy** (*β* = 0.129, *p* < 0.001)	Citak 2021 (17)
	Unplanned pregnancy (*β* = 0.096, *p* = 0.001)	Han 2022 (31)
Miscarriage	Previous miscarriage (*β* = 0.09, *p* = 0.004)	Gao 2015 (24)
Infertility	History of infertility (AOR 2.73, *p* = 0.010)	Barat 2023 (27)
Complicated pregnancy	At-risk pregnancy (*β* = 0.15, *p* < 0.01)	Taubman 2021 (33)
	History of complications in pregnancy (*β* = 0.60, *p* < 0.05) ^m^	Nguyen 2021 (26)
Own health perception	Poorer self-rated health (*β* = 2.26, *p* = 0.003)	Qiu 2019 (29)
	**Physical health** (*β* = −0.21, *p* < 0.001)	Taubman 2021 (33)
Cesarean section	No of CS (*β* = 0.071, *p* = 0.003), Previous CS experience (*β* = 0.049, *p* = 0.040)	Hou 2022 (35)
	No history of CS (*β* = 2.66, *p* = 0.011)	Zhang 2023 (36)
Healthcare services		
Antenatal visits	**Antenatal visits** (*β* = −0.125, *p* < 0.001)	Citak 2021 (17)
Doctor-patient communication	Doctor-patient communication (*β* = −0.33, *p* < 0.036)	Zhang 2023 (36)
Childbirth education or access to information		
Childbirth Class	Non-participation in childbirth preparation class (AOR 1.89, *p* = 0.040)	Beiranvand 2017 (30)
	Prenatal education (*β* = 0.093, *p* = 0.036) [No education: lower score]	Yildrim 2023 (36)
	Lack of childbirth education (AOR 2.65, *p* = 0.006)	Eroglu 2022 (28)
Smartphone applications	**Use of pregnancy-related smartphone apps** (*β* = 2.42, *p* < 0.001)	Qiu 2019 (29)
Electronic screen exposure	Screen exposure >5 h (*β* = 2.02, *p* < 0.05)	Zhang 2023 (36)
Information support	Information support (*β* = 0.13, *p* < 0.05) ^n^	Nguyen 2021 (26)
COVID-19 pandemic		
COVID-19	**Fear of COVID-19 infection** (*β* = 0.28, *p* < 0.001)	Taubman 2021 (33)
	Follows COVID-19 information (AOR = 2.23, *p* = 0.003)	Aksoy 2023 (37)

n, nullipara; m, multipara. Factors in bold have a significance value of *p* < 0.001.

### 3.1 Maternal demographics

Three studies found that maternal age was an independent factor associated with childbirth fear (17, 24, 25). Two studies demonstrated a negative association, i.e., advanced maternal age is linked with a lower level of fear (17, 24). In contrast, Huang et al. observed reduced childbirth fear among women in the younger age group (25). A study from Vietnam found that increasing age of the partner was linked to a decrease in childbirth fear (26).

Maternal education level was shown to be independently associated with FOC in four studies (17, 27–29); with three of them demonstrating an increased level of childbirth fear with higher education (27–29). Economy status posed a significant influence on maternal childbirth fear. Husband’s employment was associated with reduced odds of FOC (adjusted odd ratio, AOR 0.42, 95% CI 0.22–0.79) (27), while lack of sufficient income increased the likelihood by almost four-fold (AOR 3.70, 95% CI 1.23–11.14) (30). Study from China observed that being a part-time worker is a risk factor of FOC among multiparous (*β* = 0.141, *p* = 0.004) (31). Marital status was also an independent factor for childbirth fear, with married women reported lower level of FOC (32). Zhou et al. also reported strong association between place of residence and FOC; in which greater level of fear was found among women living in the rural area compared to the urban dwelling (32).

### 3.2 Clinical factors

Being a multipara was independently associated with reduced level of tokophobia (23, 25, 31, 33). Gestational age was also an independent predictor (25, 32), with a significant positive correlation to FOC (34). Unplanned pregnancy (17, 31), previous miscarriage (24) and history of infertility (27), were all associated with an increased risk of tokophobia. Complications in pregnancy; either in the past or present was positively associated with childbirth fear (26, 33). Pregnant women who perceived themselves in poor health also reported greater level of tokophobia (29, 33).

Hou et al. showed that the number of cesarean sections (CS) was positively associated with childbirth fear, i.e., women with two or more CS reported an increased level of FOC (*β* = 0.071, 95%CI 0.450–2.217) (35). The authors also found that the experience of previous cesarean section (moderate or severe fear) was positively related to tokophobia among Chinese women (*β* = 0.049, 95%CI 0.029–1.217) (35). In contrast, Zhang et al. observed an increased level of fear among pregnant women with no history of CS compared to individuals with previous cesarean birth (*β* = 2.66, 95% CI 0.61–4.71) (23).

### 3.3 Healthcare services

The number of antenatal visits was independently associated with reduced childbirth fear. Citak et al. observed increasing number of antenatal follow-ups was linked to a lesser degree of FOC (*β* = −0.125, *p* < 0.001) (17). Doctor-patient communication was also a significant contributor of childbirth fear with good communication was linked to lower level of tokophobia (23).

### 3.4 Childbirth education and access to information

Childbirth education was an independent predictor of FOC. Results from three studies consistently showed deficiency in prenatal preparation class was associated with an increased risk of childbirth fear (28, 30, 36).

Studies from China found positive association between the use of pregnancy related smartphone apps and prolonged screen exposure (>5 h) with greater risk of FOC (23, 29). Nguyen et al. observed a higher level of information support was linked to an increase in childbirth fear among nulliparous women in Vietnam (*β* = 0.13, *p* < 0.05) (26).

### 3.5 COVID-19 pandemic

Four studies on maternal childbirth fear were conducted during the background of COVID-19 pandemic (33, 37–39). Taubman et al. observed that women who reported fear of COVID-19 infection were more likely suffer from tokophobia (*β* = 0.28, *p* < 0.001) (33). Pregnant women who followed the COVID-19 information were twice more likely to report fear of childbirth (adjusted odd ratio, AOR 2.65, 95%CI 1.32–3.77) (37).

## 4 Discussion

The prevalence of childbirth fear differs across nations, despite similar measurement (15). Different evaluation instruments and cultural differences could explain some of the variations, albeit the exact reasons remain unknown (14, 15). More recent data demonstrated the global prevalence of severe FOC of 16% (95%CI 14–16%) with an increasing trend since 2015 (40). The prevalences of severe tokophobia in our review were higher; in the range of 0.7 to 61.5%. The variations could be attributed to the different socio-economic, political, and weaker health systems in the developing countries, compared to the more affluent Western countries (40). Developed countries like Sweden have an established multidisciplinary approach to care for women with severe FOC through the specialized clinic that is supported by experienced midwives, obstetrician, psychologist, social worker and psychiatrist (41). Continuity of care through midwifery-led models or single obstetrician follow-up throughout pregnancy which are available in Western health system (18), may partly explained the lower prevalence of tokophobia among non-Asians. Factors that characterized a population such as ethnicity, religion, beliefs, social structures, and social norms, will also influence maternal childbirth fear (42).

We found that independent association between maternal age and FOC. Studies from China and Turkey demonstrated that older mother reported lesser childbirth fear (17, 24). Other studies in this review also showed lower WDEQ-A scores among pregnant women above the age of 32, compared to the younger women (39, 43). Only one study demonstrated higher FOC among expectant in the older age group (25). Evidence from Scandinavian countries supported the link between advanced maternal age and greater childbirth fear (44, 45). Eriksson et al. reported intense FOC among primiparous women above the age of 32 (RR 2.3, 95% CI 1.6–3.5) (44). Age-related differences in the causes of childbirth fear include high-risk pregnancies in older women, and the concerns about loss of sexual enjoyment and attractiveness among younger mothers (45, 46). Lesser knowledge and experience in childbirth may also contribute toward higher FOC in young pregnant women (39).

A Vietnamese study found a significant association between the partner’s age and maternal childbirth fear. Nguyen et al. observed that a higher age of partner was associated with a lower score of FOC in mothers (*β* = −0.10, 95%CI = −0.16; −0.05) (26). This could be a reflection of tokophobia experienced by men; as evidence showed a significant positive relationship between the childbirth fear of women and their partners (*r* = 0.602, *p* < 0.001) (47). Studies from Turkey and Sweden found an increased FOC among men aged 25–35 compared to older individuals (47, 48). These men were more likely to be first-time fathers; who were at higher risk of experiencing tokophobia (AOR 1.8, 95% CI 1.2–2.6) (48). As partner support is positively correlated to maternal psychological wellbeing (*r* = 0.48, *p* < 0.001) (49), and is a mediator in the relationship between self-efficacy and childbirth fear in pregnant women (17), it is therefore important to consider the presence of tokophobia among men. Recent evidence showed that childbirth preparation for expectant fathers was associated with a more positive childbirth experience. A Swedish trial found that a psychoprophylaxis training model that focuses on the man’s role as a coach during labor, decreased the risk of frightening childbirth experiences among men with tokophobia (AOR 0.30;95% CI 0.10–0.95) (50). Bergström et al. also found that the training model which includes instruction on coaching, breathing, relaxation, and massage techniques, reduced the likelihood of feeling unprepared among expectant fathers (AOR 0.20, 95%CI 0.05–0.86) (50).

Our review showed that higher education level was an independent predictor of maternal childbirth fear. Storksen et al. made similar observation in their Norwegian study that reported increased risk of FOC among women with higher levels of education (AOR 2.1, 95% CI 1.3–3.3) (51). A large cohort study from Finland also demonstrated that higher educational level was more common among women with childbirth fear (45). Melender et al. reported that women with university level education tended to be more critical and have more negative experiences related to childbirth than controls with lower education levels (52). Individuals with higher education may seek birth-related information more actively than less educated women (51). Greater access to internet and social media among educated women lead to increased knowledge of birth complications and trauma that may exacerbate tokophobia (53). Qiu et al. suggested that educated women preferred a well-planned and structured lives. The uncontrollable aspects of childbirth that include length of labor, degree of pain and change in body shape will inevitably result in greater fear (29).

Our review demonstrated that stable and sufficient household income leads to a lesser fear of childbirth. Similarly, a study on the Danish National Birth Cohort reported a significant association between unemployment and tokophobia (AOR 1.62, 95% CI 1.35–1.93) (54). Low income and unstable finances may exacerbate the life stress among pregnant women. In contrast, a population-based study from Finland observed an increased prevalence of FOC among pregnant women of high socio-economic status (SES) regardless of parity. Raisanen et al. attributed this finding to the fact that high-risk pregnancies were common among these women, due to advanced maternal age (45).

Our observation confirmed the increased level of childbirth fear among women living in the rural area. Ogurlu et al. found that Turkish women who lived in the villages reported higher WDEQ-A scores than those living in the district areas (*p* = 0.026) (55). In urban areas, hospitalization for childbirth is advocated and the health facilities are well-resourced to deliver maternal and child healthcare. Conversely, rural health services may be deficient in professional staff and maternal care practices such as home visit, which result in greater tokophobia among expectant mothers (32, 40).

A study from Turkey demonstrated the impact of migration on FOC. Soysal et al. observed a lower level of childbirth fear among immigrants, compared to the pregnant Turkish women (*p* < 0.001). The authors also found a larger proportion of local women displaying severe form of childbirth fear than the migrant women (26.8% vs. 4.1%, *p* < 0.001) (54). In contrast, Ternström et al. reported that foreign-born women were more likely to have FOC compared to Swedish-born women (OR 2.7, 95% CI 1.7–4.0). Living in a foreign country with a different culture and language, alongside the lack of knowledge of the adopted country including civil rights and social system would probably contribute to women’s fear (56). In contrast, the migrant women in the Turkish study were mostly Syrian war refugees. Their worries would probably be lessened by the knowledge that they would give birth in a country free from conflict and with superior medical resources (53).

Our review found that being married reduced the likelihood of childbirth fear. Comparably, a recent meta-analysis of research from non-Asian countries revealed a higher prevalence of FOC among single or divorced women than those who were married or cohabited (21% vs. 15%) (40). Furthermore, data from six European countries supported the protective role of a stable civil relationship against tokophobia (AOR 0.64, 95% CI 0.45–0.87) (18). Women who received spousal support had consistently reported reduced level of FOC (25, 31, 49, 57). Spousal support during pregnancy reduces the anxiety and stress of the pregnant woman, and as that support increases, so does the expectant mother’s capacity to handle the problems she experienced during pregnancy (58). Spousal support had also been shown to be the mediator between childbirth self-efficacy (women’s confidence and ability to cope with childbirth) and FOC (17).

We found consistent evidence of reduced risk of tokophobia with multiparity. Studies from Iran and Japan also confirmed the negative correlation between childbirth fear and the number of deliveries (59, 60). A systematic review of 27 observational studies demonstrated the pooled prevalence of severe FOC in nulliparous women was higher than multiparous women (17% vs. 14%) (61). Greater tokophobia observed among the nullipara can be attributed to childbirth inexperience and fear of the unknown which include labor pain and support (62, 63). FOC among these women is also influenced by frightening information and negative birth stories from others (52). A contrast observation was made by Kaya et al. in which FOC increased significantly with the increasing number of pregnancies and parity (34). Increased tokophobia among multipara is usually related to a previous traumatic or negative birth experience (15); in parallel with the study by Hou et al. that observed greater FOC among women who experienced moderate to severe fear during their previous cesarean section (35). European data also reported a five-fold increase in the risk of childbirth fear in women with negative birth experiences (AOR 5.11, 95%CI 4.07–6.42) (18). Conversely, our review demonstrated a protective effect of multiparity against tokophobia. A mother’s prior delivery experience can boost her confidence and ability to handle challenging situations like labor and delivery (64), which will lessen her worry about the next pregnancy. Culturally, motherhood confers social value and this may encourage women to disregard their negative experiences and welcome their future pregnancy with joy and gratefulness (65).

Studies from Thailand, Turkey, and Iran demonstrated that women with unplanned pregnancy consistently reported greater WDEQ-A scores compared to individuals who planned their pregnancies (34, 42, 66). In line with our findings, a systematic review by Dencker et al. also found that unplanned pregnancy was a significant risk factor for FOC (19), which can be related to a lack of social support. Women in a stable relationship are probably more equipped to both prevent and cope with unintended pregnancies (19). Conversely, women with unplanned pregnancies reported higher FOC due to perceived inadequacy or unpreparedness for motherhood (67).

Our review found that poor maternal health is significantly associated with FOC. A similar observation was also made by Ulu et al. during the COVID-19 pandemic (39). Pregnant women are more susceptible to severe COVID-19 complications due to pregnancy-related physiological and immunity changes (68), and individuals with co-existing medical conditions are particularly vulnerable. Unsurprisingly, there was a significant positive correlation between COVID-19 fear and FOC (38, 39) and women who reported fear of COVID-19 infection were more likely to suffer from tokophobia (33).

Effective doctor-patient is important to alleviate maternal childbirth fear as evidenced by our finding. Previous research demonstrated that good communication between a physician and patient improves compliance and patient satisfaction (69). Regular antenatal care allows health professionals to offer adequate support to pregnant women including that to address fear and concerns about childbirth (34) with a positive impact on FOC reduction. A study from Iceland demonstrated that Enhanced Antenatal Care (EAC), which consists of a midwifery-led care model that combines one-to-one and group antenatal care is more effective in reducing tokophobia than standard care (Cohen’s d = −0.21) (70). The EAC model provides the usual antenatal appointments focusing on screening and physical assessment, alongside group sessions that allow extensive discussion about pregnancy, birth, and parenthood with midwives and other expecting parents. The authors concluded that the high satisfaction among women in the EAC group was likely due to increased social support, education, continuity of care, and personal relationship with midwives which women value in antenatal care services (70).

Insufficient knowledge of labor has been associated with increased childbirth fear (34, 71). Prenatal education had been shown not only to improve maternal knowledge, but also reduce anxiety regarding delivery (72) and fear of childbirth (73). Learning about childbirth brought comfort to some women as it helped them prepare for labor (74) and recognize how uncommon certain birth complications were (75). However, having too much information especially contradicting ones might elevate the fear of childbirth (74–76). This may explain the positive correlation between FOC and the level of information support among nullipara.

The internet has allowed women to access and share various information on delivery process, coping with labor pain alongside experience on pregnancy, birth and infant care (29, 77). Poor quality resources such as false or conflicting information, and unfavorable stories are more likely to have a negative impact on women’s perception of childbirth, resulting in increased FOC (29, 52). Unsurprisingly, prolonged screen exposure and the use of smartphone apps predisposed expectant mothers to greater childbirth fear. Women with extended internet use were more likely to access the forum web pages with a high amount of traumatic birth stories that could produce unrealistic picture of childbirth (78). Recent evidence indicated that exposure to electronic screens for more than 5 h per day in pregnant women was a risk factor for depressive symptoms (79) that in turn were associated with childbirth fear (32).

Healthcare professionals therefore play a vital role in providing the relevant advice and guidance to expectant mothers on trustworthy information sources, and timely emotional support to diminish their anxiety during the pregnancy period (80).

### 4.1 Strengths and limitations

Our paper is the first to systematically review studies that were exclusive to the Asian population. This review discussed the independent factors associated with maternal childbirth fear, that were derived from multivariable regression analysis. This review provided more insight into Asian women’s fear of childbirth, which is different from that of the more commonly researched European and Scandinavian populations.

Our review has several limitations. First off, the results may not accurately reflect the Asian nations as data from other countries in Southeast Asia, the Middle East, and the Indian subcontinent is lacking. Secondly, the cross-sectional design of the studies in our review prevents the establishment of causal relationship between the variables. Thirdly, due to differing assessment tools and cutoffs, we were unable to conduct a meta-analysis. The fourth point, qualitative studies that may provide deeper cultural insights into childbirth fear among Asian women, were not included in our review as we focused on quantitative research. The fifth point, as we excluded studies on Asian immigrants in non-Asian countries, we might have missed the factors influenced by the diasporic experiences of childbirth fear. Lastly, given the heterogeneity of the population in terms of ethnicity, religion, economic development, and social structure; results interpretation should be done with particular caution.

## 5 Conclusion

The greater FOC prevalence among Asian women is attributed to various factors including the country’s resources, socio-demographics, and cultural beliefs. Financial and relationship stability are important to reduce maternal childbirth fear. Medical conditions affecting maternal health or pregnancy will lead to increased FOC. Prenatal education is vital to impart the necessary knowledge to expectant mothers. However, conflicting information and facts overload may result in higher FOC, especially among first-time mothers. Health professionals play vital roles in addressing childbirth fear through adequate health provision, effective communication, childbirth education, and guidance on trustworthy information. Therefore, initiatives to address tokophobia among Asian women should include culturally appropriate communication training among healthcare providers, alongside locally adapted antenatal programs and mental health support for expectant mothers.

## Data Availability

The original contributions presented in the study are included in the article/supplementary material, further inquiries can be directed to the corresponding authors.
